# Breast cancer screening motivation and behaviours of women aged over 75 years: a scoping review

**DOI:** 10.1186/s12905-024-03094-z

**Published:** 2024-04-24

**Authors:** Virginia Dickson-Swift, Joanne Adams, Evelien Spelten, Irene Blackberry, Carlene Wilson, Eva Yuen

**Affiliations:** 1 Violet Vines Centre for Rural Health Research, La Trobe Rural Health School, La Trobe University, P.O. Box 199, Bendigo, VIC 3552 Australia; 2 Care Economy Research Institute, La Trobe University, Wodonga, Australia; 3 Olivia Newton-John Cancer Wellness and Research Centre, Austin Health, Melbourne, Australia; 4 Melbourne School of Population and Global Health, Melbourne University, Melbourne, Australia; 5 School of Psychology and Public Health, La Trobe University, Bundoora, Australia; 6 Institute for Health Transformation, Deakin University, Burwood, Australia; 7 Centre for Quality and Patient Safety, Monash Health Partnership, Monash Health, Clayton, Australia; 8 School of Psychology and Public Health, La Trobe University, Bundoora, Australia

**Keywords:** Behaviour, Breast cancer, Mammography, Motivation, Older women, Screening, Scoping review

## Abstract

**Background:**

This scoping review aimed to identify and present the evidence describing key motivations for breast cancer screening among women aged ≥ 75 years. Few of the internationally available guidelines recommend continued biennial screening for this age group. Some suggest ongoing screening is unnecessary or should be determined on individual health status and life expectancy. Recent research has shown that despite recommendations regarding screening, older women continue to hold positive attitudes to breast screening and participate when the opportunity is available.

**Methods:**

All original research articles that address motivation, intention and/or participation in screening for breast cancer among women aged ≥ 75 years were considered for inclusion. These included articles reporting on women who use public and private breast cancer screening services and those who do not use screening services (i.e., non-screeners).

The Joanna Briggs Institute (JBI) methodology for scoping reviews was used to guide this review. A comprehensive search strategy was developed with the assistance of a specialist librarian to access selected databases including: the Cumulative Index to Nursing and Allied Health Literature (CINAHL), Medline, Web of Science and PsychInfo. The review was restricted to original research studies published since 2009, available in English and focusing on high-income countries (as defined by the World Bank). Title and abstract screening, followed by an assessment of full-text studies against the inclusion criteria was completed by at least two reviewers. Data relating to key motivations, screening intention and behaviour were extracted, and a thematic analysis of study findings undertaken.

**Results:**

A total of fourteen (14) studies were included in the review. Thematic analysis resulted in identification of three themes from included studies highlighting that decisions about screening were influenced by: knowledge of the benefits and harms of screening and their relationship to age; underlying attitudes to the importance of cancer screening in women's lives; and use of decision aids to improve knowledge and guide decision-making.

**Conclusion:**

The results of this review provide a comprehensive overview of current knowledge regarding the motivations and screening behaviour of older women about breast cancer screening which may inform policy development.

## Introduction

Breast cancer is now the most commonly diagnosed cancer in the world overtaking lung cancer in 2021 [1]. Across the globe, breast cancer contributed to 25.8% of the total number of new cases of cancer diagnosed in 2020 [2] and accounts for a high disease burden for women [3]. Screening for breast cancer is an effective means of detecting early-stage cancer and has been shown to significantly improve survival rates [4]. A recent systematic review of international screening guidelines found that most countries recommend that women have biennial mammograms between the ages of 40–70 years [5] with some recommending that there should be no upper age limit [6–12] and others suggesting that benefits of continued screening for women over 75 are not clear [13–15].

Some guidelines suggest that the decision to end screening should be determined based on the individual health status of the woman, their life expectancy and current health issues [5, 16, 17]. This is because the benefits of mammography screening may be limited after 7 years due to existing comorbidities and limited life expectancy [18–21], with some jurisdictions recommending breast cancer screening for women ≥ 75 years only when life expectancy is estimated as at least 7–10 years [22]. Others have argued that decisions about continuing with screening mammography should depend on individual patient risk and health management preferences [23]. This decision is likely facilitated by a discussion between a health care provider and patient about the harms and benefits of screening outside the recommended ages [24, 25]. While mammography may enable early detection of breast cancer, it is clear that false-positive results and overdiagnosis^1^ may occur. Studies have estimated that up to 25% of breast cancer cases in the general population may be over diagnosed [26–28].

The risk of being diagnosed with breast cancer increases with age and approximately 80% of new cases of breast cancer in high-income countries are in women over the age of 50 [29]. The average age of first diagnosis of breast cancer in high income countries is comparable to that of Australian women which is now 61 years [2, 4, 29]. Studies show that women aged ≥ 75 years generally have positive attitudes to mammography screening and report high levels of perceived benefits including early detection of breast cancer and a desire to stay healthy as they age [21, 30–32]. Some women aged over 74 participate, or plan to participate, in screening despite recommendations from health professionals and government guidelines advising against it [33]. Results of a recent review found that knowledge of the recommended guidelines and the potential harms of screening are limited and many older women believed that the benefits of continued screening outweighed the risks [30].

Very few studies have been undertaken to understand the motivations of women to screen or to establish screening participation rates among women aged ≥ 75 and older. This is surprising given that increasing age is recognised as a key risk factor for the development of breast cancer, and that screening is offered in many locations around the world every two years up until 74 years. The importance of this topic is high given the ambiguity around best practice for participation beyond 74 years. A preliminary search of Open Science Framework, PROSPERO, Cochrane Database of Systematic Reviews and JBI Evidence Synthesis in May 2022 did not locate any reviews on this topic.

This scoping review has allowed for the mapping of a broad range of research to explore the breadth and depth of the literature, summarize the evidence and identify knowledge gaps [34, 35]. This information has supported the development of a comprehensive overview of current knowledge of motivations of women to screen and screening participation rates among women outside the targeted age of many international screening programs.

## Materials and methods

### Research question

The research question for this scoping review was developed by applying the Population—Concept—Context (PCC) framework [36]. The current review addresses the research question “What research has been undertaken in high-income countries (context) exploring the key motivations to screen for breast cancer and screening participation (concepts) among women ≥ 75 years of age (population)?

### Eligibility criteria

#### Participants

Women aged ≥ 75 years were the key population. Specifically, motivations to screen and screening intention and behaviour and the variables that discriminate those who screen from those who do not (non-screeners) were utilised as the key predictors and outcomes respectively.

#### Concept

From a conceptual perspective it was considered that motivation led to behaviour, therefore articles that described motivation and corresponding behaviour were considered. These included articles reporting on women who use public (government funded) and private (fee for service) breast cancer screening services and those who do not use screening services (i.e., non-screeners).

#### Context

The scope included high-income countries using the World Bank definition [37]. These countries have broadly similar health systems and opportunities for breast cancer screening in both public and private settings.

### Types of sources

All studies reporting original research in peer-reviewed journals from January 2009 were eligible for inclusion, regardless of design. This date was selected due to an evaluation undertaken for BreastScreen Australia recommending expansion of the age group to include 70–74-year-old women [38]. This date was also indicative of international debate regarding breast cancer screening effectiveness at this time [39, 40]. Reviews were also included, regardless of type—scoping, systematic, or narrative. Only sources published in English and available through the University’s extensive research holdings were eligible for inclusion. Ineligible materials were conference abstracts, letters to the editor, editorials, opinion pieces, commentaries, newspaper articles, dissertations and theses.

This scoping review was registered with the Open Science Framework database (https://osf.io/fd3eh) and followed Joanna Briggs Institute (JBI) methodology for scoping reviews [35, 36]. Although ethics approval is not required for scoping reviews the broader study was approved by the University Ethics Committee (approval number HEC 21249).

### Search strategy

A pilot search strategy was developed in consultation with an expert health librarian and tested in MEDLINE (OVID) and conducted on 3 June 2022. Articles from this pilot search were compared with seminal articles previously identified by the members of the team and used to refine the search terms. The search terms were then searched as both keywords and subject headings (e.g., MeSH) in the titles and abstracts and Boolean operators employed. A full MEDLINE search was then carried out by the librarian (see Table 1). This search strategy was adapted for use in each of the following databases: Cumulative Index to Nursing and Allied Health Literature (CINAHL), Medical Literature Analysis and Retrieval System Online (MEDLINE), Web of Science and PsychInfo databases. The references of included studies have been hand-searched to identify any additional evidence sources.

**Table 1:** Search strategy developed for MEDLINE (conducted 3 June 2022)

Search ID#	Search Terms	Search Notes	Results
S1	Mammography/	MeSH	32229
S2	Mammogra*		44024
S3	(breast ADJ2 screen*)		11700
S4	1 OR 2 OR 3		48125
S5	Aged/	MeSH	3352052
S6	“Aged, 80 and over”/	MeSH	1006581
S7	((old* OR elder* OR senior* OR geriatric* OR ageing OR aging) ADJ1 (women* OR woman* OR female))		253779
S8	“over 75*” OR “aged 75” OR “75 year*” OR “75 plus” OR 75 +		528574
S9	OR/5–8		3967581
S10	Early detection of cancer/	MeSH	33895
S11	Mass screening/	MeSH	113577
S12	“early detection”		103374
S13	Screen*		965921
S14	(detect* ADJ2 cancer*)		61097
S15	OR/10–14		1046292
S16	4 and 9 and 15		10373
S17	Limit 16 to (English language and yr = ”2009-current”)		4158

* denotes a range of alternate endings for the word

### Study/source of evidence selection

Following the search, all identified citations were collated and uploaded into EndNote v.X20 (Clarivate Analytics, PA, USA) and duplicates removed. The resulting articles were then imported into Covidence – Cochrane’s systematic review management software [41]. Duplicates were removed once importation was complete, and title and abstract screening was undertaken against the eligibility criteria. A sample of 25 articles were assessed by all reviewers to ensure reliability in the application of the inclusion and exclusion criteria. Team discussion was used to ensure consistent application. The Covidence software supports blind reviewing with two reviewers required at each screening phase. Potentially relevant sources were retrieved in full text and were assessed against the inclusion criteria by two independent reviewers. Conflicts were flagged within the software which allows the team to discuss those that have disagreements until a consensus was reached. Reasons for exclusion of studies at full text were recorded and reported in the scoping review. The Preferred Reporting Items of Systematic Reviews extension for scoping reviews (PRISMA-ScR) checklist was used to guide the reporting of the review [42] and all stages were documented using the PRISMA-ScR flow chart [42].

### Data extraction

A data extraction form was created in Covidence and used to extract study characteristics and to confirm the study’s relevance. This included specific details such as article author/s, title, year of publication, country, aim, population, setting, data collection methods and key findings relevant to the review question. The draft extraction form was modified as needed during the data extraction process.

### Data analysis and presentation

Extracted data were summarised in tabular format (see Table 2). Consistent with the guidelines for the effective reporting of scoping reviews [43] and the JBI framework [35] the final stage of the review included thematic analysis of the key findings of the included studies. Study findings were imported into QSR NVivo with coding of each line of text. Descriptive codes reflected key aspects of the included studies related to the motivations and behaviours of women > 75 years about breast cancer screening.

**Table 2:** Details of included studies

Author Publication Year Country	Sample	Design	Main Outcomes	Themes	Main findings
Beckmeyer 2020 USA [45]	13 women aged 75 +	Quantitative Pilot test decision aid and pre/post survey design	Receipt of mammography screening at 18 months Knowledge of the benefits and harms of mammography, decisional conflict around screening (including 5 subscales), preferred decision-making role, whether participants discussed mammography or HS with their PCP, and changes in screening intention, acceptability of the DA	3	Prior to intervention, all participants planned to have a screening mammogram in the next year. Post-intervention, 5 of the 8 women (62.5%) indicated they intended to continue to receive mammography; however, 3 participants planned to get them less often. When asked whether they thought their physician would want them to get a mammogram, 80% said “yes” on pre-test; this figure decreased to 62.5%. This pilot study suggests that the use of a decision-aid may result in fewer women ≥ 75 years old continuing to screen for breast cancer as of the 7 women who at pre-test thought they would get an-other mammogram, only 4 individuals at post-test said that they intended to get one in the next year. This is consistent with a previous-study of the DA showing a decrease in intent-to- screen from 82% pre-test to 56% post-test on post-test
Brotzman 2022 USA [46]	19 women aged 71–93	Qualitative description	Experiences, beliefs, and opinions about screening mammography in relation to aging and health	1,2,	Three main themes emerged: (1) older women typically perceive mammograms as a positive, beneficial, and routine component of care; (2) participation in routine mammography is reinforced by factors at interpersonal, provider, and healthcare system levels; and (3) older women do not endorse discontinuation of screening mammography due to advancing age or poor health, but some may be receptive to reducing screening frequency. Only a few older women reported having discussed mammography cessation or the potential harms of screening with their providers. A few women reported they would insist on receiving mammography even without a provider recommendation
Cadet 2021 USA [48]	43 aged 75–89	Quantitative Pre/post-test trial	Knowledge of benefits and harms, decisional conflict, decision making role and acceptability	1,3	Receipt of the DA significantly affected knowledge of mammography’s benefits and harms (pre-test(M = 3.75,SD = 1.05) to post-test (M = 4.42,SD = 1.19), p = .03).Receipt of the DA did not significantly affect decisional conflict (pre-test M = 3.10,SD = .97) to post-test M = 3.23,SD = 1.02,p = .71)- higher scores = lower decisional conflict. The majority of the women (97%) indicated that the DA was helpful
Cadet 2021 USA [49]	18 women aged 75–89	Qualitative	Knowledge, acceptability of the DA	1,3	Findings indicate that women in this study lacked knowledge and understanding that one can decide on mammography screening based on their personal values. Women were enthusiastic about screening based on an interest in taking care of themselves but rely on their providers for health care decisions. Overall, most women found the DA helpful and would recommend the use of the DA
Cadet 2021 USA [47]	283 aged 75–89	Quantitative Secondary analysis	Association between women’s educational attainment and their knowledge of the benefits and harms of mammography Decisional conflict, change in screening intention, discussions with PCP and receipt of screening	3	Regardless of educational attainment, 87.2% of the 283 women found the DA helpful. Women with lower educational attainment were less likely to understand all the DA’s content (46.3% vs 67.5%; P < .001), had less knowledge of the benefits and harms of mammography (adjusted mean ± standard error knowledge score, 7.1 ± 0.3 vs 8.1 ± 0.3; P < .001), and were less likely to lower screening intentions (adjusted percentage, 11.4% vs 19.4%; P = .01). Receipt of screening did not differ by educational attainment
Collins 2010 UK [23]	26 interviews and 479 questionnaires	Mixed methods	Breast screening knowledge and uptake Breast screening awareness and behaviour, views about screening and preferences for screening	1,2	Over half (52.9%) of the respondents were unaware that they could request a mammography by voluntary self-referral and were unaware how to arrange this. Most (81.5%) had not attended breast screening since turning 70 years. Most (75.6%) felt screening was beneficial and would attend if invited. Most (90.1%) felt screening should be offered to all women regardless of age or health. The overwhelming view across both the interview and questionnaire data was that breast screening should be offered to all women indefinitely and regardless of age, health status or fitness (90.1%, CI: 87.0–92.6%). There was a strong preference for unrestricted screening with 42.9%, indicating their preference for automatic recall extended indefinitely regard-less of age or health status. Almost three in four women surveyed (74.1%) indicated a preference for a postal reminder letter every 3 years
Eisinger 2011 France [56]	136 women 75 years and over as a subset of a larger sample	Quantitative survey	Screening intention	2	For 136 women aged 75 years and over, who were breast cancer-free and who had undergone a mammography at least once in their lifetime, 62 (51%) had done so during the previous two years. Of these 62 women, 37 (60%) intended to pursue screening in the future; of the 60 women who had never undergone a mammography, 27 (36%) intended to do so in the future. Future intentions regarding screening varied significantly [odds ratio (OR) = 2.6, 95% confidence interval (CI) = 1.2–5.4]. However, no sociodemographic differences were observed between screened and unscreened women regarding level of education, income, health risk behaviour (smoking, alcohol consumption), knowledge about the importance and the process of screening, or psychological features (fear of the test, fear of the results, fear of the disease, trust in screening impact)
Gray 2018 USA [50]	Not reported at individual level	Quantitative using data from national cross-sectional survey	Impact of social interaction on screening Education, income and employment	1	For all women, evidence was found of social interactions associated with individual’s education, employment, and poor health. In addition, number of age-group-specific social multipliers was found. The strongest evidence of spill over in mammography was found for women ages 75 and older. Policy makers should be aware that, in the presence of a social multiplier, the value of any type of screening intervention is higher than the one that would be measured at the individual-level
Hoover 2019 USA [51]	31 women aged 75 +	Qualitative descriptive	Knowledge of benefits and harms of screening Information provision about over-diagnosis	1,2	Participants wanted to hear about the benefits and harms of screening mammography, including over diagnosis. Participants requested information be communicated via physicians or other healthcare providers, included in brochures/pamphlets, and presented outside of clinical settings (e.g., in senior groups). Results were consistent regardless of participants’ age, race/ethnicity, or education. Findings revealed that older women desire information about the benefits and harms of screening mammography and would prefer to learn this information through discussions with healthcare providers and multiple other formats
Housten 2018 USA [33]	11 women aged 75 +	Qualitative	Reasons for discontinuation of screening	1	All women expressed a strong intention to continue screening. Based on the hypothetical physician recommendations, intentions to continue screening appeared to remain strong. They did not envision a change in their health status that would lead them to discontinue screening and were sceptical of expert/government recommendations. There were no differences observed according to age, race/ethnicity, or education
Salzman 2020 USA [52]	24 women aged 75 +	Qualitative	Knowledge about screening, discussions with providers,	1,3	Most participants (75%) reported familiarity with current breast cancer screening guidelines. Twenty-nine percent reported prior discussions with providers about continuing breast cancer screenings. Sixty percent did not need assistance completing DAs while 40% did. 66.7% found the decision aids “very helpful” in reflecting their breast cancer screening thoughts; 58.3% had no preference regarding either decision aid version. 75% of participants were willing to complete the decision aid before a provider visit. Participants equally preferred a health educator or provider facilitating discussion of breast cancer screening harms and benefits and potential cessation
Schoenborn 2021 USA [53]	283 women aged 75 +	Quantitative as part of a larger RCT (pre/post visit with PCP)	Intention to be screened and receipt of mammography screening	1, 3	From pre to post visit with their PCP, 21.7% of women lowered their intentions to be screened, 7.9% increased their intentions to be screened, and 70.4% did not change. Lower screening intention is associated with lower breast cancer screening rates among older women, suggesting that screening intention is a reasonable proximal outcome for interventions aimed at reducing over screening in older women
Schonberg 2014 USA [55]	45 women aged 75 +	Quantitative Pre/post-test trial design	Knowledge about mammography Decisional conflict, screening intention, decision-making role, acceptability and discussions with providers and receipt of screening	3	The median age of participants was 79 years. Comparison of post-test results with pre-test results demonstrated 2 findings. First, knowledge of the benefits and risks of screening improved (P < .001). Second, fewer participants intended to be screened (56% [25 of 45] afterward compared with 82% [37 of 45] before, P = .03). Decisional conflict declined but not significantly (p = .10). In the following 6 months, 53% (24 of 45) of participants had a primary care physician note that documented the discussion of the risks and benefits of screening compared with 11% (5 of 45) in the previous 5 years (P < .001). While 84% (36 of 43) had been screened within 2 years of participating, 60% (26 of 43) were screened within 15 months after participating (2 years since their last mammogram) (P = .01). Overall, 93% (42 of 45) found the DA helpful
Schonberg 2020 USA (54)	283 women aged 75 + that received the DA	Quantitative RCT	Receipt of mammography screening at 18 months Knowledge of the benefits and harms of mammography, decisional conflict around screening (including 5 subscales), discussed mammography or HS with their PCP, and changes in screening intention. Preferred decision-making role, whether participants	3	Receipt of the decision aid before a visit with their clinician led to women 75 years and older being more knowledgeable about mammography screening, having more discussions with their primary care physician about screening, and fewer women being screened

## Results

**Fig. 1:**
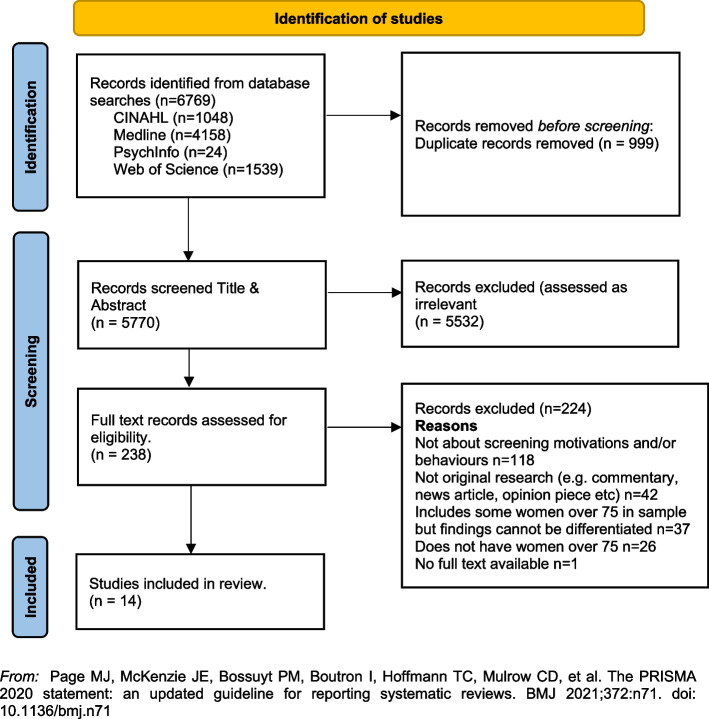
PRISMA Flowchart. From: Page MJ, McKenzie JE, Bossuyt PM, Boutron I, Hoffmann TC, Mulrow CD, et al. The PRISMA 2020 statement: an updated guideline for reporting systematic reviews. BMJ 2021;372:n71. https://doi.org/10.1136/bmj.n71

In line with the reporting requirements for scoping reviews the search results for this review are presented in Fig. 1 [44].

A total of fourteen [14] studies were included in the review with studies from the following countries, US *n* = 12 [33, 45–55], UK *n* = 1 [23] and France *n* = 1 [56]. Sample sizes varied, with most containing fewer than 50 women (*n* = 8) [33, 45, 46, 48, 51, 52, 55]. Two had larger samples including a French study with 136 women (a sub-set of a larger sample) [56], and one mixed method study in the UK with a sample of 26 women undertaking interviews and 479 women completing surveys [23]. One study did not report exact numbers [50]. Three studies [47, 53, 54] were undertaken by a group of researchers based in the US utilising the same sample of women, however each of the papers focused on different primary outcomes. The samples in the included studies were recruited from a range of locations including primary medical care clinics, specialist medical clinics, University affiliated medical clinics, community-based health centres and community outreach clinics [47, 53, 54].

Data collection methods varied and included: quantitative (*n* = 8), qualitative (*n* = 5) and mixed methods (*n* = 1). A range of data collection tools and research designs were utilised; pre/post, pilot and cross-sectional surveys, interviews, and secondary analysis of existing data sets. Seven studies focused on the use of a Decision Aids (DAs), either in original or modified form, developed by Schonberg et al. [55] as a tool to increase knowledge about the harms and benefits of screening for older women [45, 47–49, 52, 54, 55]. Three studies focused on intention to screen [33, 53, 56], two on knowledge of, and attitudes to, screening [23, 46], one on information needs relating to risks and benefits of screening discontinuation [51], and one on perceptions about discontinuation of screening and impact of social interactions on screening [50].

The three themes developed from the analysis of the included studies highlighted that decisions about screening were primarily influenced by: (1) knowledge of the benefits and harms of screening and their relationship to age; (2) underlying attitudes to the importance of cancer screening in women's lives; and (3) exposure to decision aids designed to facilitate informed decision-making. Each of these themes will be presented below drawing on the key findings of the appropriate studies. The full dataset of extracted data can be found in Table 2.

### Knowledge of the benefits and harms of screening ≥ 75 years

The decision to participate in routine mammography is influenced by individual differences in cognition and affect, interpersonal relationships, provider characteristics, and healthcare system variables. Women typically perceive mammograms as a positive, beneficial and routine component of care [46] and an important aspect of taking care of themselves [23, 46, 49]. One qualitative study undertaken in the US showed that few women had discussed mammography cessation or the potential harms of screening with their health care providers and some women reported they would insist on receiving mammography even without a provider recommendation to continue screening [46].

Studies suggested that ageing itself, and even poor health, were not seen as reasonable reasons for screening cessation. For many women, guidance from a health care provider was deemed the most important influence on decision-making [46]. Preferences for communication about risk and benefits were varied with one study reporting women would like to learn more about harms and risks and recommended that this information be communicated via physicians or other healthcare providers, included in brochures/pamphlets, and presented outside of clinical settings (e.g., in community-based seniors groups) [51]. Others reported that women were sometimes sceptical of expert and government recommendations [33] although some were happy to participate in discussions with health educators or care providers about breast cancer screening harms and benefits and potential cessation [52].

### Underlying attitudes to the importance of cancer screening at and beyond 75 years

Included studies varied in describing the importance of screening, with some attitudes based on past attendance and some based on future intentions to screen. Three studies reported findings indicating that some women intended to continue screening after 75 years of age [23, 45, 46], with one study in the UK reporting that women supported an extension of the automatic recall indefinitely, regardless of age or health status. In this study, failure to invite older women to screen was interpreted as age discrimination [23]. The desire to continue screening beyond 75 was also highlighted in a study from France that found that 60% of the women (*n* = 136 aged ≥ 75) intended to pursue screening in the future, and 27 women aged ≥ 75, who had never undergone mammography previously (36%), intended to do so in the future [56]. In this same study, intentions to screen varied significantly [56]. There were no sociodemographic differences observed between screened and unscreened women with regard to level of education, income, health risk behaviour (smoking, alcohol consumption), knowledge about the importance and the process of screening, or psychological features (fear of the test, fear of the results, fear of the disease, trust in screening impact) [56]. Further analysis showed that three items were statistically correlated with a higher rate of attendance at screening: (1) screening was initiated by a physician; (2) the women had a consultation with a gynaecologist during the past 12 months; and (3) the women had already undergone at least five screening mammograms. Analysis highlighted that although average income, level of education, psychological features or other types of health risk behaviours did not impact screening intention, having a mammogram previously impacted likelihood of ongoing screening. There was no information provided that explained why women who had not previously undergone screening might do so in the future.

A mixed methods study in the UK reported similar findings [23]. Utilising interviews (*n* = 26) and questionnaires (*n* = 479) with women ≥ 70 years (median age 75 years) the overwhelming result (90.1%) was that breast screening should be offered to all women indefinitely regardless of age, health status or fitness [23], and that many older women were keen to continue screening. Both the interview and survey data confirmed women were uncertain about eligibility for breast screening. The survey data showed that just over half the women (52.9%) were unaware that they could request mammography or knew how to access it. Key reasons for screening discontinuation were not being invited for screening (52.1%) and not knowing about self-referral (35.1%).

Women reported that not being invited to continue screening sent messages that screening was no longer important or required for this age group [23]. Almost two thirds of the women completing the survey (61.6%) said they would forget to attend screening without an invitation. Other reasons for screening discontinuation included transport difficulties (25%) and not wishing to burden family members (24.7%). By contrast, other studies have reported that women do not endorse discontinuation of screening mammography due to advancing age or poor health, but some may be receptive to reducing screening frequency on recommendation from their health care provider [46, 51].

### Use of Decision Aids (DAs) to improve knowledge and guide screening decision-making

Many women reported poor knowledge about the harms and benefits of screening with studies identifying an important role for DAs. These aids have been shown to be effective in improving knowledge of the harms and benefits of screening [45, 54, 55] including for women with low educational attainment; as compared to women with high educational attainment [47]. DAs can increase knowledge about screening [47, 49] and may decrease the intention to continue screening after the recommended age [45, 52, 54]. They can be used by primary care providers to support a conversation about breast screening intention and reasons for discontinuing screening. In one pilot study undertaken in the US using a DA, 5 of the 8 women (62.5%) indicated they intended to continue to receive mammography; however, 3 participants planned to get them less often [45]. When asked whether they thought their physician would want them to get a mammogram, 80% said “yes” on pre-test; this figure decreased to 62.5% after exposure to the DA. This pilot study suggests that the use of a decision-aid may result in fewer women ≥ 75 years old continuing to screen for breast cancer [45].

Similar findings were evident in two studies drawing on the same data undertaken in the US [48, 53]. Using a larger sample (*n* = 283), women’s intentions to screen prior to a visit with their primary care provider and then again after exposure to the DA were compared. Results showed that 21.7% of women reduced their intention to be screened, 7.9% increased their intentions to be screened, and 70.4% did not change. Compared to those who had no change or increased their screening intentions, women who had a decrease in screening intention were significantly less likely to receive screening after 18 months. Generally, studies have shown that women aged 75 and older find DAs acceptable and helpful [47–49, 55] and using them had the potential to impact on a women’s intention to screen [55].

Cadet and colleagues [49] explored the impact of educational attainment on the use of DAs. Results highlight that education moderates the utility of these aids; women with lower educational attainment were less likely to understand all the DA’s content (46.3% vs 67.5%; P < 0.001); had less knowledge of the benefits and harms of mammography (adjusted mean ± standard error knowledge score, 7.1 ± 0.3 vs 8.1 ± 0.3; p < 0.001); and were less likely to have their screening intentions impacted (adjusted percentage, 11.4% vs 19.4%; *p* = 0.01).

## Discussion

This scoping review summarises current knowledge regarding motivations and screening behaviours of women over 75 years. The findings suggest that awareness of the importance of breast cancer screening among women aged ≥ 75 years is high [23, 46, 49] and that many women wish to continue screening regardless of perceived health status or age. This highlights the importance of focusing on motivation and screening behaviours and the multiple factors that influence ongoing participation in breast screening programs.

The generally high regard attributed to screening among women aged ≥ 75 years presents a complex challenge for health professionals who are focused on potential harm (from available national and international guidelines) in ongoing screening for women beyond age 75 [18, 20, 57]. Included studies highlight that many women relied on the advice of health care providers regarding the benefits and harms when making the decision to continue breast screening [46, 51, 52], however there were some that did not [33]. Having a previous pattern of screening was noted as being more significant to ongoing intention than any other identified socio-demographic feature [56]. This is perhaps because women will not readily forgo health care practices that they have always considered important and that retain ongoing importance for the broader population.

For those women who had discontinued screening after the age of 74 it was apparent that the rationale for doing so was not often based on choice or receipt of information, but rather on factors that impact decision-making in relation to screening. These included no longer receiving an invitation to attend, transport difficulties and not wanting to be a burden on relatives or friends [23, 46, 51]. Ongoing receipt of invitations to screen was an important aspect of maintaining a capacity to choose [23]. This was particularly important for those women who had been regular screeners.

Women over 75 require more information to make decisions regarding screening [23, 52, 54, 55], however health care providers must also be aware that the element of choice is important for older women. Having a capacity to choose avoids any notion of discrimination based on age, health status, gender or sociodemographic difference and acknowledges the importance of women retaining control over their health [23]. It was apparent that some women would choose to continue screening at a reduced frequency if this option was available and that women should have access to information facilitating self-referral [23, 45, 46, 51, 56].

Decision-making regarding ongoing breast cancer screening has been facilitated via the use of Decision Aids (DAs) within clinical settings [54, 55]. While some studies suggest that women will make a decision regardless of health status, the use of DAs has impacted women’s decision to screen. While this may have limited benefit for those of lower educational attainment [48] they have been effective in improving knowledge relating to harms and benefits of screening particularly where they have been used to support a conversation with women about the value of screening [54–56].

Women have identified challenges in engaging in conversations with health care providers regarding ongoing screening, because providers frequently draw on projections of life expectancy and over-diagnosis [17, 51]. As a result, these conversations about screening after age 75 years often do not occur [46]. It is likely that health providers may need more support and guidance in leading these conversations. This may be through the use of DAs or standardised checklists. It may be possible to incorporate these within existing health preventive measures for this age group. The potential for advice regarding ongoing breast cancer screening to be available outside of clinical settings may provide important pathways for conversations with women regarding health choices. Provision of information and advice in settings such as community based seniors groups [51] offers a potential platform to broaden conversations and align sources of information, not only with health professionals but amongst women themselves. This may help to address any misconception regarding eligibility and access to services [23]. It may also be aligned with other health promotion and lifestyle messages provided to this age group.

### Limitations of the review

The searches that formed the basis of this review were carried in June 2022. Although the search was comprehensive, we have only captured those studies that were published in the included databases from 2009. There may have been other studies published outside of these periods. We also limited the search to studies published in English with full-text availability.

The emphasis of a scoping review is on comprehensive coverage and synthesis of the key findings, rather than on a particular standard of evidence and, consequently a quality assessment of the included studies was not undertaken. This has resulted in the inclusion of a wide range of study designs and data collection methods. It is important to note that three studies included in the review drew on the same sample of women (283 over > 75)[49, 53, 54]. The results of this review provide valuable insights into motivations and behaviours for breast cancer screening for older women, however they should be interpreted with caution given the specific methodological and geographical limitations.

## Conclusion and recommendations

This scoping review highlighted a range of key motivations and behaviours in relation to breast cancer screening for women ≥ 75 years of age. The results provide some insight into how decisions about screening continuation after 74 are made and how informed decision-making can be supported. Specifically, this review supports the following suggestions for further research and policy direction:

1. Further research regarding breast cancer screening motivations and behaviours for women over 75 would provide valuable insight for health providers delivering services to women in this age group.
2. Health providers may benefit from the broader use of decision aids or structured checklists to guide conversations with women over 75 regarding ongoing health promotion/preventive measures.
3. Providing health-based information in non-clinical settings frequented by women in this age group may provide a broader reach of information and facilitate choices. This may help to reduce any perception of discrimination based on age, health status or socio-demographic factors.

## Data Availability

All data generated or analysed during this study is included in this published article (see Table 2 above).

## Footnotes

1 Cancer Australia, in their 2014 position statement, define “overdiagnosis” in the following way. ‘’Overdiagnosis’ from breast screening does not refer to error or misdiagnosis, but rather refers to breast cancer diagnosed by screening that would not otherwise have been diagnosed during a woman’s lifetime. “Overdiagnosis” includes all instances where cancers detected through screening (ductal carcinoma in situ or invasive breast cancer) might never have progressed to become symptomatic during a woman’s life, i.e., cancer that would not have been detected in the absence of screening. It is not possible to precisely predict at diagnosis, to which cancers overdiagnosis would apply.” (accessed 22.^nd^ August 2022; https://www.canceraustralia.gov.au/resources/position-statements/overdiagnosis-mammographic-screening).
